# Supplementary material for: Inappropriate antibiotic use in the COVID-19 era: Factors associated with inappropriate prescribing and secondary complications. Analysis of the registry SEMI-COVID
Source: PLoS One. 2021 May 11;16(5):e0251340. doi: 10.1371/journal.pone.0251340 (PMC8112666; doi:10.1371/journal.pone.0251340)
Supplement: S1 Annex — (DOCX) [file pone.0251340.s002.docx]

S1 Annex. Examples of local protocols that included recommendation for macrolide use due to its supposed antiviral and immunomodulatory effect.


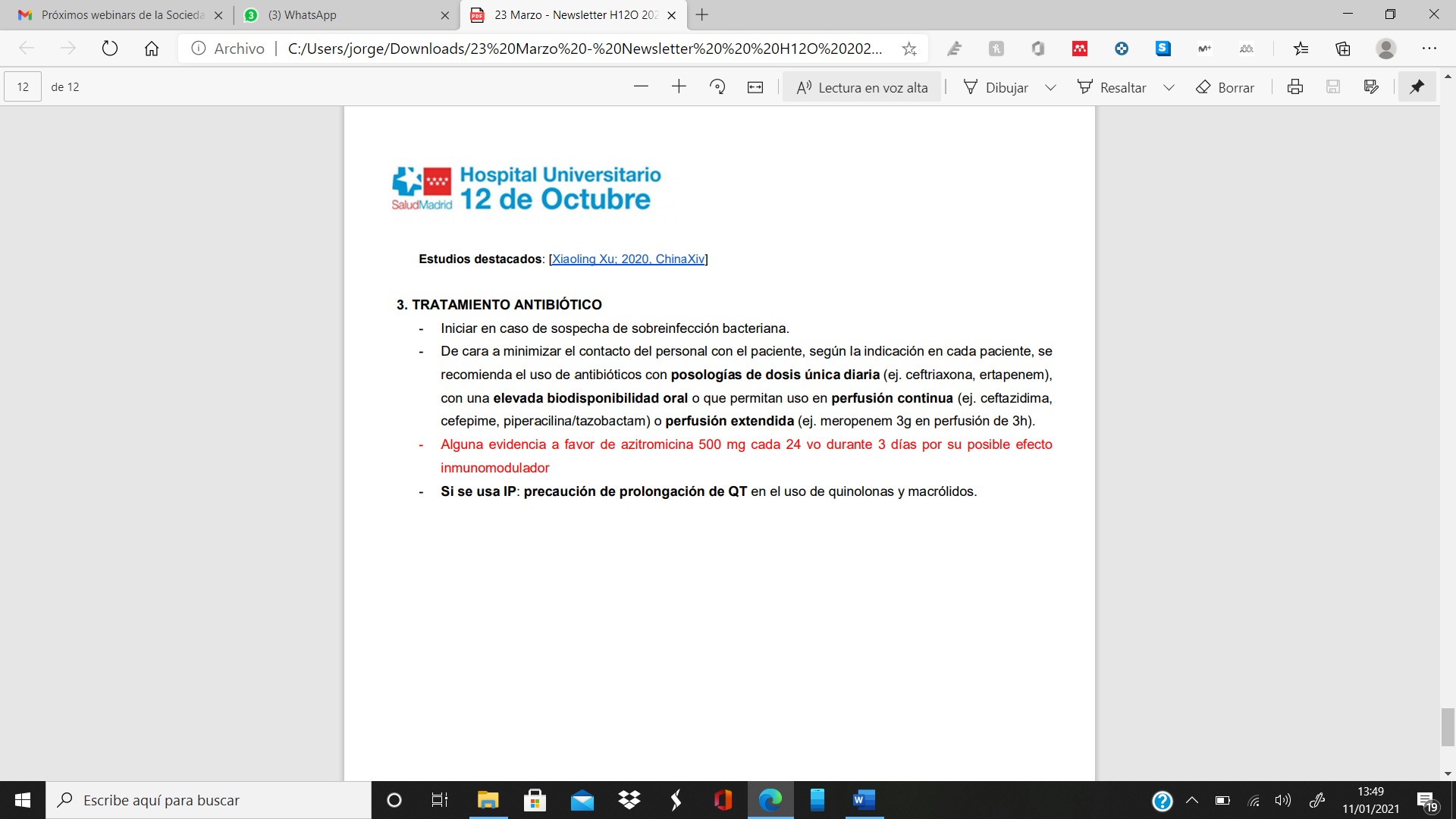


1: 12 de Octubre Local Protocol, with date of March 23, 2020


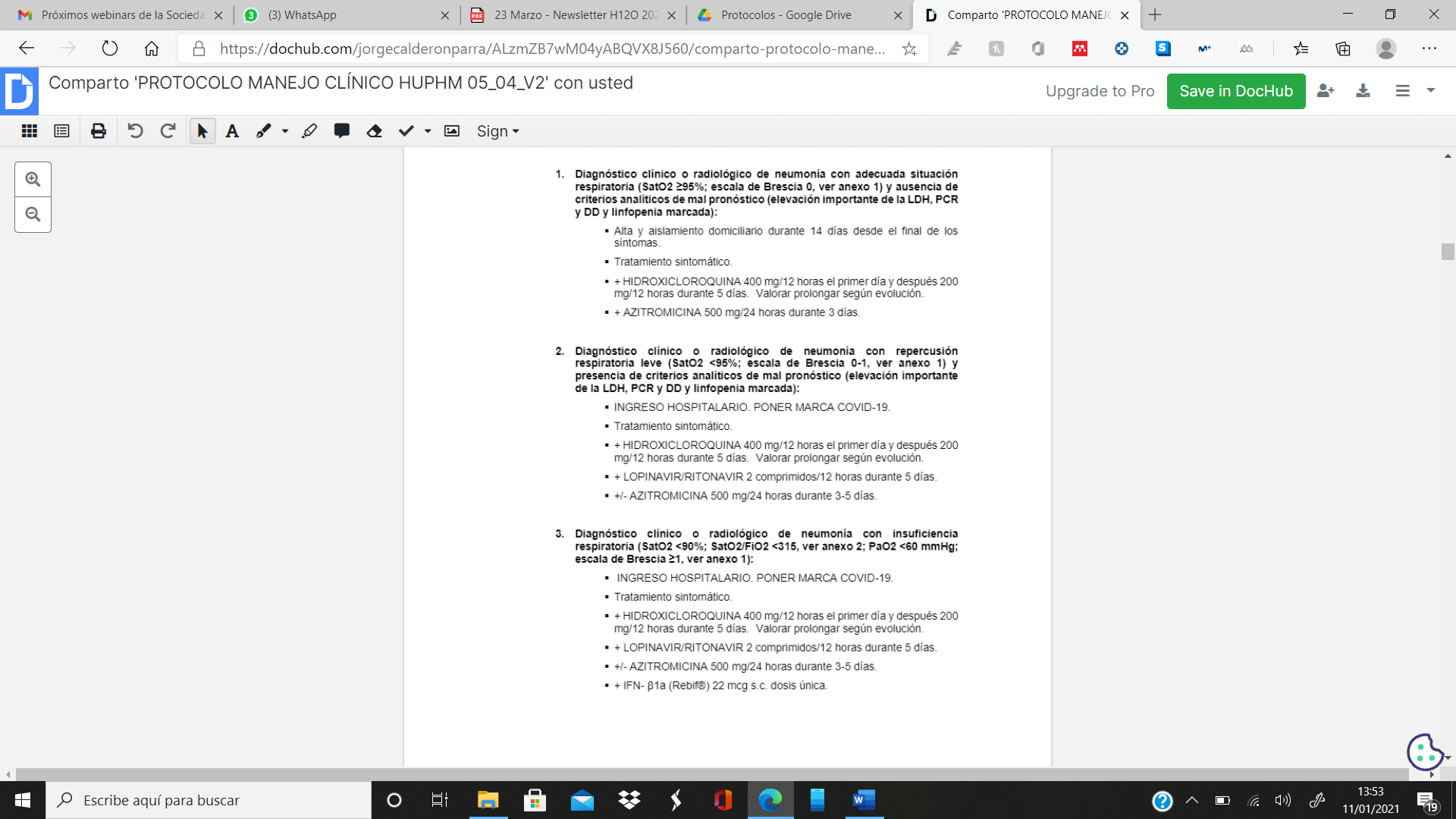


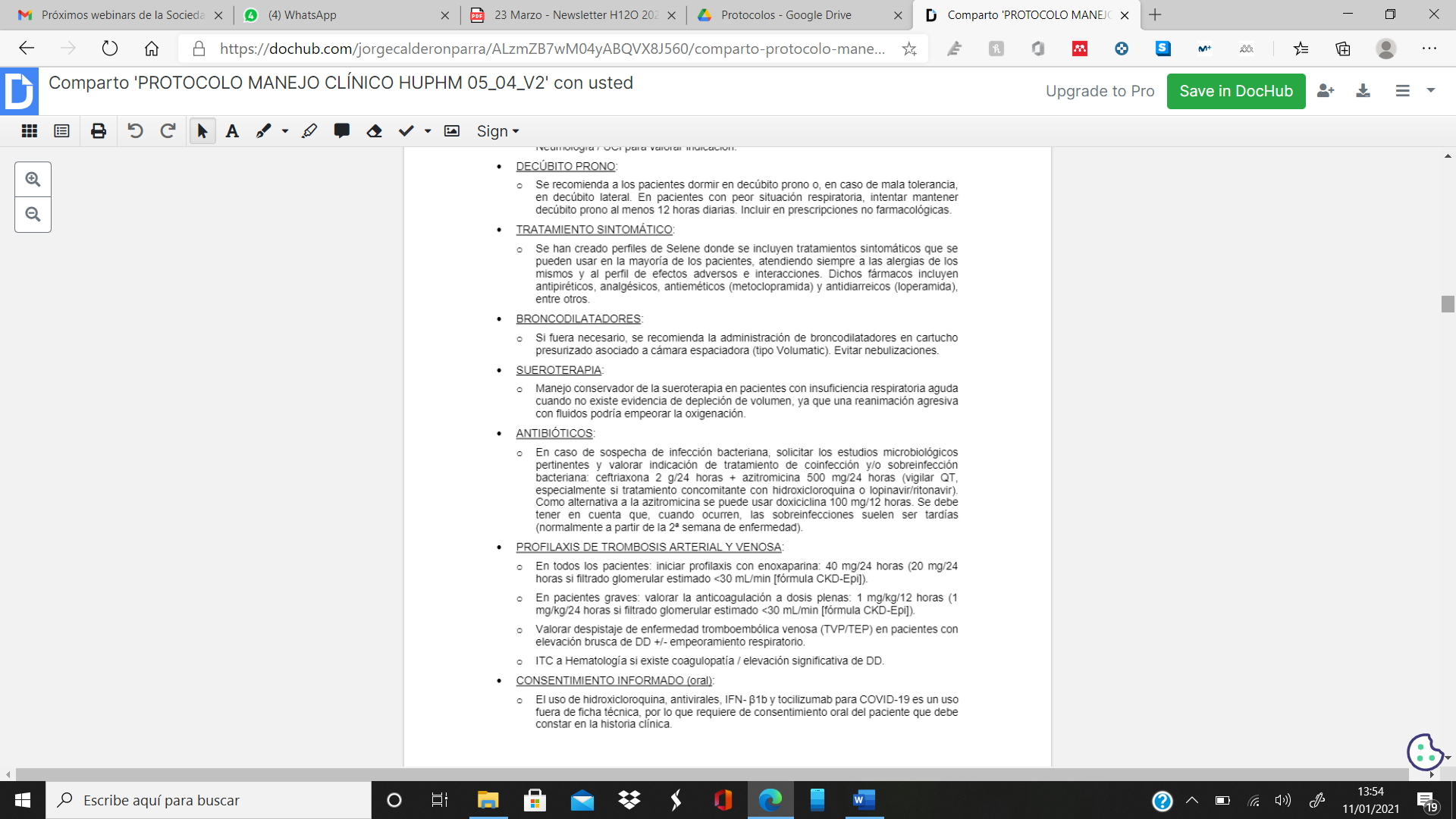


2: Puerta de Hierro Local Protocol, with date April 5, 2020, in which there is a recommendation for the use of azithromycin for its immunomodulatory effect, while there is a recommendation against the use of other antibiotics in the absence of suspected bacterial infection.
